# MMP-7 Serum and Tissue Levels Are Associated with Poor Survival in Platinum-Treated Bladder Cancer Patients

**DOI:** 10.3390/diagnostics11010048

**Published:** 2020-12-31

**Authors:** Tibor Szarvas, Michèle J. Hoffmann, Csilla Olah, Eszter Szekely, Andras Kiss, Jochen Hess, Stephan Tschirdewahn, Boris Hadaschik, Vera Grotheer, Peter Nyirady, Anita Csizmarik, Melinda Varadi, Henning Reis

**Affiliations:** 1 Department of Urology, University of Duisburg-Essen, 45147 Essen, Germany; Csilla.Olah@uk-essen.de (C.O.); Jochen.Hess@uk-essen.de (J.H.); Stephan.Tschirdewahn@uk-essen.de (S.T.); Boris.Hadaschik@uk-essen.de (B.H.); 2 Department of Urology, Semmelweis University, 1089 Budapest, Hungary; nyirady.peter@med.semmelweis-univ.hu (P.N.); csizmarik.anita@med.semmelweis-univ.hu (A.C.); varadi.melinda_rita@med.semmelweis-univ.hu (M.V.); 3 Department of Urology, Medical Faculty, Heinrich-Heine-University Duesseldorf, 40225 Duesseldorf, Germany; Michele.Hoffmann@uni-duesseldorf.de; 4 2nd Department of Pathology, Semmelweis University, 1091 Budapest, Hungary; szekely.eszter@med.semmelweis-univ.hu (E.S.); kiss.andras@med.semmelweis-univ.hu (A.K.); 5 Department of Orthopedics and Trauma Surgery, Medical Faculty, Heinrich-Heine-University Duesseldorf, 40225 Duesseldorf, Germany; Vera.Grotheer@uni-duesseldorf.de; 6 Institute of Pathology, University Hospital Essen, University of Duisburg-Essen, 45147 Essen, Germany; Henning.Reis@uk-essen.de

**Keywords:** bladder cancer, MMP-7, cisplatin, chemotherapy, metastasis, prognosis

## Abstract

Chemotherapy resistance is a main cause of therapeutic failure and death in bladder cancer. With the approval of immune checkpoint inhibitors, prediction of platinum treatment became of great clinical importance. Matrix metalloproteinase-7 (MMP-7) was shown to be involved in cisplatin resistance. Therefore, tissue and circulating MMP-7 levels were evaluated in 124 bladder cancer patients who received postoperative platinum-based chemotherapy. Tissue MMP-7 levels were analyzed by immunohistochemistry in 72 formalin-fixed, paraffin-embedded chemo-naïve tumor samples, while MMP-7 serum concentrations were determined in 132 serum samples of an independent cohort of 52 patients. MMP-7 tissue and serum levels were correlated with clinicopathological and follow-up data. MMP-7 gene expression was determined by RT-qPCR in 20 urothelial cancer cell lines and two non-malignant urothelial cell lines. MMP-7 was overexpressed in RT-112 and T-24 cells by stable transfection, to assess its functional involvement in platinum sensitivity. High MMP-7 tissue expression and pretreatment serum concentrations were independently associated with poor overall survival (tissue HR = 2.296, 95%CI = 1.235–4.268 and *p* = 0.009; serum HR = 2.743, 95%CI = 1.258–5.984 and *p* = 0.011). Therefore, MMP-7 tissue and serum analysis may help to optimize therapeutic decisions. Stable overexpression in RT-112 and T-24 cells did not affect platinum sensitivity.

## 1. Introduction

Urothelial bladder carcinoma (bladder cancer (BC)) represents a significant health burden with 550,000 new cases worldwide and over 200,000 deaths annually [1]. Although about 70% of all BCs are diagnosed in a non-muscle invasive stage, 10–15% of patients will develop muscle-invasive (MIBC), locally advanced and/or metastatic disease. In addition, 10% of newly diagnosed patients have locally advanced and/or metastatic disease. For these patients, cisplatin-based combination chemotherapy with MVAC (methotrexate, vinblastine, doxorubicin and cisplatin) or GC (gemcitabine and cisplatin) has been the only treatment of choice for over 30 years. Even though BC is considered a cisplatin-sensitive disease, only 40–60% of patients show a radiographic response [2]. Recently, targeted FGFR2/3 and immune checkpoint inhibitors became available for patients with MIBC that had progressed during or following platinum-containing chemotherapy and later also for patients who are ineligible for platinum therapy [3,4,5,6]. With the approval of novel potentially effective therapies for patients with progressed muscle-invasive BC, the prediction of platinum therapy became of great clinical importance. Since clinical and pathological parameters are unable to distinguish between platinum sensitive and resistant BCs, much effort has been made to identify novel therapy-predicting markers [7]. Gene-expression-based molecular subtypes were reported to be associated with survival in platinum-treated MIBC patients [8]. However, other authors could not confirm this observation [9]. Therefore, prediction of survival for patients who receive platinum-based chemotherapy remains an unmet clinical need.

Formerly, Ansell et al. performed a comparative genome-wide transcript profiling, using microarray analysis to find genes differentially expressed in cisplatin-sensitive versus -resistant head-and-neck squamous cell carcinoma (HNSCC) cells. The identified 781 genes were further selected by using in silico methods, and, finally, 20 key regulator genes were verified in 25 HNSCC cell lines. This multistep analysis revealed matrix metalloproteinase-7 (MMP-7) as a predictive marker for chemotherapy resistance [10]. Accordingly, a growing body of evidence supported that MMP-7 is involved in platinum resistance by allowing tumor cells to escape from chemotherapy-induced apoptosis [11,12,13,14,15,16]. These promising results and the urgent need for chemotherapy-predictive markers led us to evaluate whether MMP-7 tissue and serum levels are associated with tumor progression and survival in patients with advanced BC treated with platinum-based chemotherapy. 

## 2. Materials and Methods 

### 2.1. Patients

The study included two independent cohorts, with a total number of 124 BC patients. For both cohorts, patients’ characteristics are given in Table 1.

The first “IHC (immunohistochemistry) cohort” included 72 patients with available chemo-naïve formalin-fixed and paraffin-embedded (FFPE) tumor tissue who underwent postoperative platinum treatment between January/2004 and March/2010 in our institution (*n* = 29) or participated in a phase II, prospective, multicenter, randomized, double-blinded trial (SUSE, AB 31/05, RUTT 204) (*n* = 43).

The second “ELISA (enzyme-linked immunosorbent assay) cohort” consisted of 52 BC patients with available serum samples who received platinum treatment between January/2010 and December/2017 at the Department of Urology, University Hospital of Essen (*n* = 16), and the Department of Urology of Semmelweis University of Budapest (*n* = 36). Serum samples were collected postoperatively prior to the first platinum treatment (at baseline). In addition, for 21 patients an overall number of 80 serum samples collected at predefined time points during chemotherapy were available for monitoring analysis.

Inclusion criteria for this study were as follows: (1) locally advanced or metastatic urothelial BC, (2) no prior chemotherapy (3) and written informed consent. The primary endpoint was overall survival (OS) and secondary endpoint was progression-free survival (PFS). 

The study was performed according to the ethical standards of the Helsinki Declaration, and the study protocol was approved by the ethical board of the University of Duisburg-Essen and Semmelweis University of Budapest (15-6400-BO/(May 2015)) and TUKEB 55/2014 (November 2013).

### 2.2. MMP-7 Immunohistochemistry (IHC)

IHC was performed on 4 µm thick paraffin sections of chemo-naive BC specimens using a mouse monoclonal antibody against MMP-7 (JL07, Santa Cruz Biotechnology, dilution 1:75) as described previously [17]. Staining intensity was scored as 0, 1, 2 or 3, corresponding to negative, weak, moderate and strong intensities, while percentage score was defined as 0–10% = 0 points, 11–50% = 1 point, 51–80% = 2 points and 81–100% = 3 points. Finally, a score was calculated by multiplying the intensity score by the percentage score (results from 0 to 9 points). High MMP-7 expression was considered as IHC-score higher than 3. 

### 2.3. MMP-7 ELISA

Serum MMP-7 levels were determined by sandwich enzyme-linked immunosorbent assay, using a Quantikine ELISA kit from R&D Systems (Wiesbaden, Germany) according to the manufacturer’s instructions.

### 2.4. Cell Culture

Twenty different urothelial carcinoma cells (UCCs) and two immortalized urothelial cell lines were cultured as described in the Supplementary Materials [18,19].

### 2.5. Gene Expression Analysis

Details on RNA extraction, reverse transcription and quantitative polymerase chain reaction (qRT-PCR) from cultured cells are described in the Supplementary Methods. 

### 2.6. Stable Overexpression of MMP-7 and Analysis of Cisplatin Sensitivity

For stable overexpression, we used the expression plasmid pcDNA3-GFP-MMP-7, which was a gift from Steven Johnson (Addgene plasmid #11989; http://n2t.net/addgene:11989; RRID: Addgene_11989). Details on this method and viability measurements are provided in the Supplementary Materials. 

### 2.7. In Silico Analysis of MMP-7 Gene Expression in the TCGA Dataset

The freely available raw TCGA (The Cancer Genome Atlas) II transcriptome database of 405 MIBC samples (http://www.cbioportal.org/study?id=blca_tcga) was used (1) to determine the association between *MMP-7* gene expression and MIBC molecular subtypes, (2) to assess prognostic value of *MMP-7* gene expression in a mostly non-chemo treated MIBC cohort and (3) to correlate *MMP-7* gene expression with stromal and immune gene signatures [20]. These signature scores were determined by using the ESTIMATE algorithm, which calculated the stromal and immune specific transcriptomic profile of BC patients from the TCGA cohort, thereby estimating the tumor purity [21]. Then we divided patients into a low (*n* = 203) and a high (*n* = 202) signature score group and compared MMP-7 expression between these groups. 

### 2.8. Statistical Analysis

Associations of MMP-7 immunostaining with clinicopathological parameters were evaluated by using the chi-square test. We used the non-parametric two-sided Wilcoxon rank sum test for independent group comparisons. Univariate OS and PFS analyses were done by using the Kaplan–Meier log-rank test and univariate Cox analysis. For multiple analysis, the Cox proportional hazards regression model was used. The Mann–Whitney test was used to examine the correlation between *MMP-7* gene expression and molecular subtypes, and stromal and immune signature. In all tests, *p*-values < 0.05 were considered statistically significant. All statistical analyses were done with the SPSS software package (SPSS v26.0, IBM, Armonk, NY, USA).

## 3. Results

Patients’ follow-up characteristics for the serum ELISA and tissue IHC cohorts are given in Table 1. For the ELISA cohort, the median age of patients was 65 years, (range: 41–81). In total, 38 of 52 (73%) patients were male. Moreover, 43 patients (83%) had an Eastern Co-Operative Oncology Group (ECOG) performance status of 0, and 9 (17%) had a PS > 0. In 41 cases, pathological tumor stage was available (pT1: *n* = 1, pT2: *n* = 9, pT3: *n* = 21, pT4: *n* = 10). Nine (17%) patients had visceral metastases, while 34 (65%) had lymph node metastases alone (Table 1). The median OS for all patients was 16.5 months (range: 2–101) and PFS was 13.3 months (range: 1–101). 

For the IHC cohort, the median age of patients was 64 years, ranging from 37 to 90 years. In all, 50 of 72 (69%) patients were male, and 22 (31%) were female. Moreover, 37 patients (51%) had an ECOG performance status of 0, and 35 (49%) had a PS > 0. In 54 cases, pathological tumor stage was available (pT1: *n* = 1, pT2: *n* = 16, pT3: *n* = 24, pT4: *n* = 13). Thirty-six (50%) patients had visceral metastases, while 41 (72%) had lymph node metastases (Table 1). The median OS for all patients was 8.7 months (range: 1–102), and PFS was 5.1 months (range: 1–102).

### 3.1. Correlation of MMP-7 Levels with Clinicopathological Parameters 

**Figure 1:**
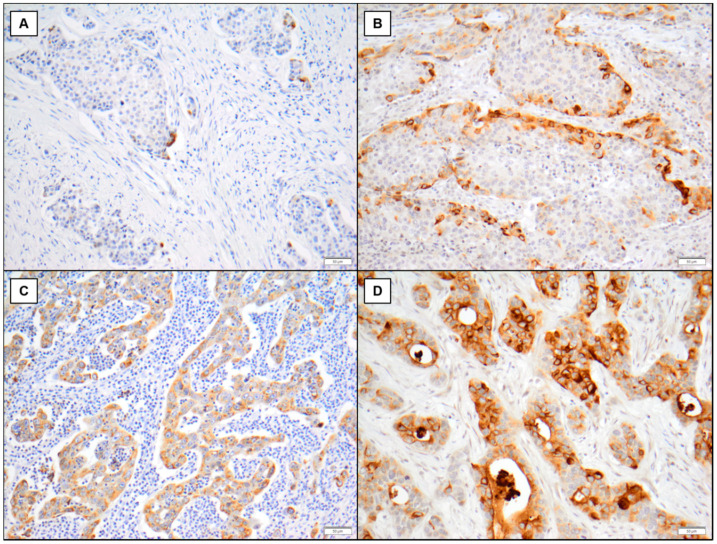
Different staining patterns in MMP-7 immunohistochemistry. (**A**,**B**) Tumor cells only show strong immunopositivity at the tumor–stroma interface. (**A**) Only scattered cells are positive. (**B**) MMP-7-protein is expressed in a nearly linear fashion in tumor cells at the tumor–stroma border. (**C**,**D**) Nearly all tumor cells exhibit a diffuse and moderate to strong MMP-7-expression with strong intratumoral neutrophil infiltration in (**C**) and only very few immune cells in the tumor stroma in (**D**). However, in both (**C**,**D**) the tumor cells in the trabecular and/or glandular infiltrations are all in close proximity to the tumor–stroma interface. All 200×.

MMP-7 staining showed a diffuse, fine granular pattern and was predominantly localized in the cytoplasm of tumor cells (Figure 1). MMP-7 was expressed by the tumor cells and, in many cases, focally localized to tumor cells at the tumor–stroma interface, while in some cases, a weak MMP-7 expression could be observed with a rather homogeneous distribution within the tumor (Figure 1). High MMP-7 expression was found in 19 of 72 samples (26%). We did not find any correlation between MMP-7 tissue expression/serum concentration and clinicopathological parameters (Table 2).

### 3.2. Correlation of MMP-7 Levels and Survival

Univariate analysis revealed that the presence of visceral metastasis was associated with shorter OS and PFS in the ELISA cohort (*p* = 0.013 and *p* = 0.002, respectively) and tended to be associated with poor OS (*p* = 0.104) in the IHC cohort (Table 3).

**Figure 2:**
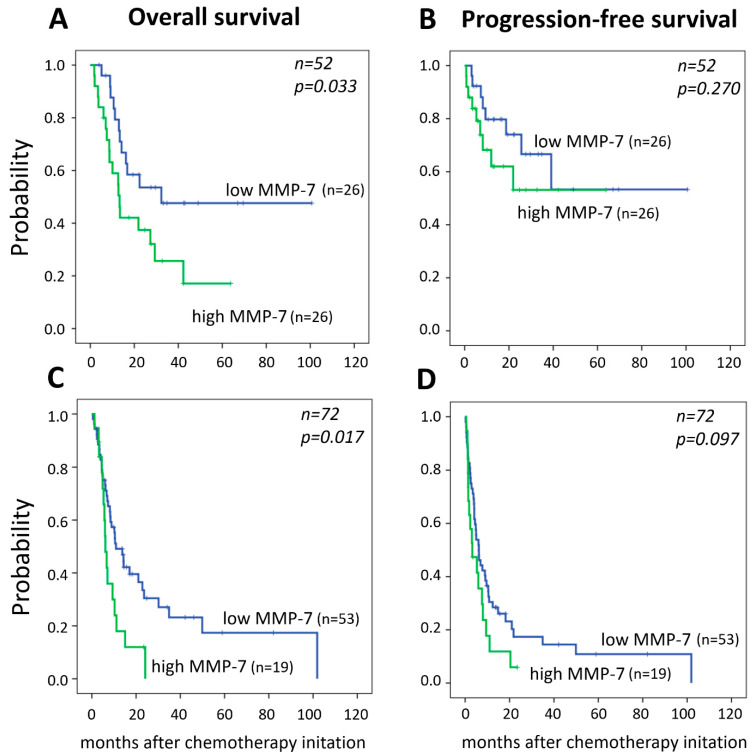
Kaplan–Meier curves of overall and progression-free survival stratified by MMP-7 (**A**,**B**) serum concentration (>10 ng/mL); (**C**,**D**) high MMP-7 tissue expression (score > 3).

High MMP-7 tissue protein expressions and serum concentrations were significantly correlated with poor OS (*p* = 0.017 and *p* = 0.033, respectively) (Figure 2). Accordingly, the median OS and PFS were shorter (12.8 and 8.2 months) for patients with high MMP-7 serum levels (>10.0 ng/mL), compared to those of with low MMP-7 levels (20.8 and 19.1 months). Similarly, in the IHC cohort, the median OS and PFS were shorter (5.8 and 3.1 months) for patients with high MMP-7 tissue expression (10.4 and 6.2 months). 

In the IHC cohort, multivariate analysis revealed that patients’ baseline ECOG performance status and high MMP-7 expression were independent risk-factors for OS (*p* = 0.002 and *p* = 0.009, respectively) and PFS (*p* = 0.003 and *p* = 0.049, respectively) (Table 4). Similarly, in the ELISA cohort, high pretreatment MMP-7 serum levels were independently associated with shorter OS (*p* = 0.011). In addition, the presence of visceral metastases was an independent risk factor for PFS (*p* = 0.009). 

### 3.3. Changes of MMP-7 Serum Levels during Platinum-Based Chemotherapy

In 21 patients with available monitoring samples taken during platinum treatment, we did not find any correlation between changes of MMP-7 levels and patients’ survival (Supplementary Materials Figure S1A–D). In 10 of 21 patients, MMP-7 levels increased; in three patients, it decreased; and in eight patients, it remained unchanged during platinum treatment. Patients with progression tended to have higher baseline MMP-7 serum levels that further increased after chemotherapy cycles. However, we did not find any significant difference between these groups regarding their OS and PFS. 

### 3.4. Functional Analyses of MMP-7 in BC Cell Lines

**Figure 3:**
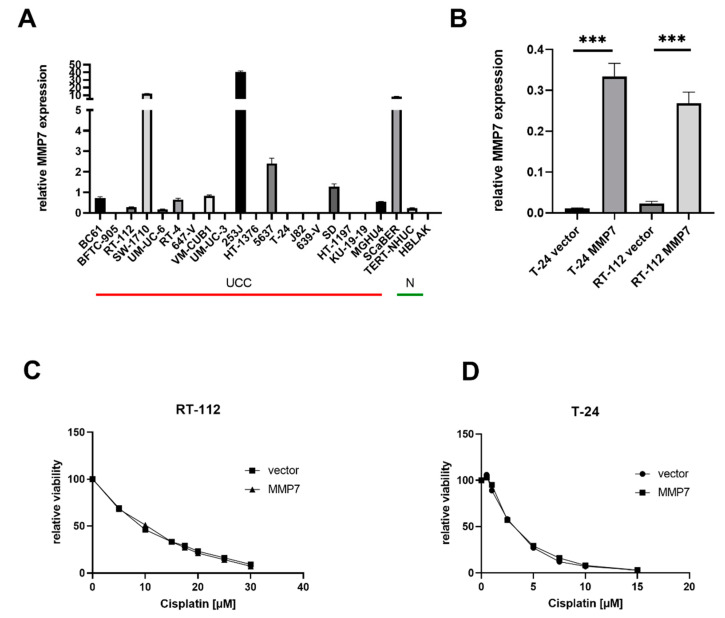
*MMP-7* gene expression in urothelial carcinoma cells (UCCs), control cells and transfected cells and its correlation with cisplatin sensitivity. (**A**) Expression of *MMP-7* was measured by RT-qPCR in triplicates and normalized to the reference gene *TATA-Box binding protein* (*TBP*). Expression of 20 bladder cancer (BC) cell lines (UCCs, red line) was compared to two normal control cell lines (N, green line). *** marks statistical significance with *p* < 0.001. (**B**) Verification of stable overexpression of MMP-7 in UCC T-24 and RT-112 by RT-qPCR. Expression of *MMP-7* was measured by RT-qPCR in quadruplicates (technical duplicates from two biological replicates) and normalized to the reference gene TBP. (**C**,**D**) Cisplatin sensitivity of *MMP-7* overexpressing UCCs. Cell viability was measured in quadruplicates by CellTiter Glo assay, 72 h after treatment, with indicated cisplatin doses. Viability of MMP-7 overexpressing cells was normalized to vector control cells. One representative result of three independent experiments is displayed.

To analyze the functional impact of MMP-7 on cisplatin sensitivity of BC cell lines, we determined MMP-7 gene expression in a set of 20 UCCs and two normal control cell lines by qRT-PCR (Figure 3A). MMP-7 was heterogeneously expressed across cell lines, ranging from 0.002 to 40.5 relative expression units, as compared to a mean expression of 0.11 in normal control cells. We observed significant overexpression in 10/20 UCCs, which was particularly high in SW1710, 253J and SCaBER cells.

For functional analysis by stable overexpression, we focused on UCCs with low endogenous MMP-7 expression. RT-112 and T-24 had no or low endogenous MMP-7 expression and represented well-differentiated (RT-112) and less-differentiated (T-24) tumors. In previous work, these cell lines belonged to a set of four BC lines used to establish long-term Cisplatin-resistant sublines. Thus, we knew IC50 doses for cisplatin for various UCCs from previous work [22], and we chose RT-112 as a cell line with a high baseline IC50 (IC50 72 h 10 µM) and T-24 as one with a low baseline IC50 (IC50 72 h 2 µM). Overexpression following stable transfection was confirmed by RT-qPCR. MMP-7 was 30- (T24) and 11-fold (RT-112) overexpressed, as compared to empty vector cells, respectively (Figure 3B).

Subsequently, stably overexpressing and vector cells were treated with seven different doses of cisplatin, for 72 h, to determine cisplatin sensitivity. However, cell viability analyses did not reveal any changes in cisplatin sensitivity by MMP-7 overexpression (Figure 3C,D).

### 3.5. In Silico Analysis of MMP-7 Gene Expression in the TCGA II Cohort

**Figure 4:**
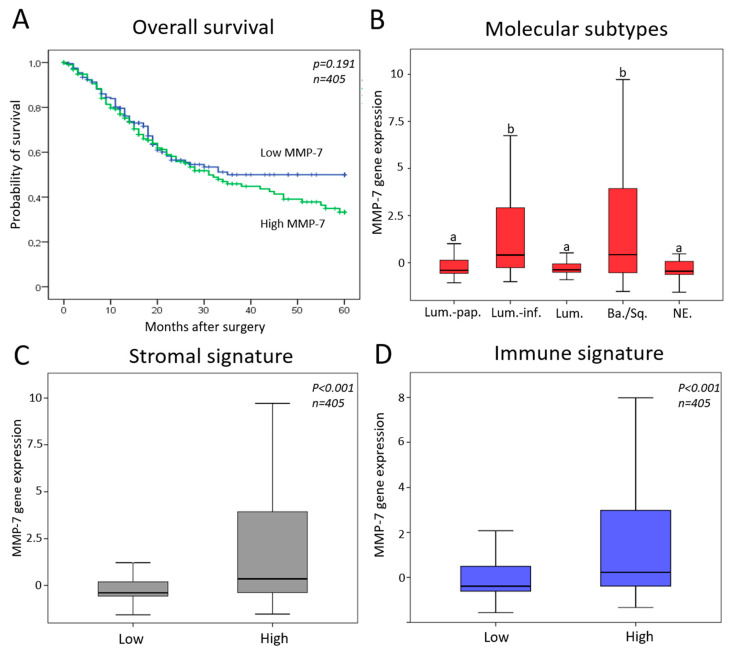
*MMP-7* gene expression in the TCGA cohort (**A**) Kaplan–Meier plots for patients’ overall survival with low (*n* = 203) and high (*n* = 202) *MMP-7* expression. (**B**) *MMP-7* gene expression was significantly associated with TCGA II subtypes. Various letters present significant differences (*p* < 0.001). Molecular subtypes signed with “a” have significantly lower MMP-7 expression, as compared to subtypes signed with “b”. (**C**) *MMP-7* gene expression was significantly higher in samples with a high stromal signature score. (**D**) *MMP-7* gene expression was significantly higher in samples with a high immune signature score. *MMP-7* gene expression levels were normalized to TBP, according to the ΔCt method. Lum.-pap.—luminal-papillary, Lum.-Inf.—luminal-infiltrated, Lum.—luminal, Ba./Sq.—Basal/Squamous, Ne.—Neuronal subtypes.

We found that *MMP-7* gene expression did not correlate significantly with overall survival in the TCGA II cohort (*p* = 0.191) (Figure 4A). Thus, we correlated *MMP-7* gene expressions with molecular subtypes of the TCGA dataset. *MMP-7* gene expression was significantly higher in luminal-infiltrated and basal/squamous subtypes (*p* < 0.001) (Figure 4B). We also revealed a positive association between high *MMP-7* gene expression and high stromal (*p* < 0.001) and immune signature (*p* < 0.001) (Figure 4C,D). 

## 4. Discussion

In the present study, we demonstrated, for the first time, that high MMP-7 tissue expressions and serum concentrations are independent risk factors for early disease progression and poor survival in patients treated with cisplatin-based chemotherapy. However, the functional involvement of MMP-7 in platinum resistance could not be proven by our in vitro analyses. In addition, our data suggest the importance of tumor–stroma interaction in the regulation of MMP-7.

Patients with similar clinical and pathological characteristics may show different responses to platinum-based chemotherapy. Chemotherapy resistance greatly influences therapy effectiveness and is considered to be one of the main causes of individual differences in therapeutic sensitivity. Formerly, studies in various tumors revealed MMP-7 as a potential marker of chemoresistance. Liu et al. described a significant correlation between MMP-7 and Bcl-2 in tumor samples of lung adenocarcinoma patients [13]. We found high pretreatment MMP-7 serum levels to be independently associated with poor response and shorter OS of castration-resistant prostate cancer patients who received docetaxel chemotherapy [23,24]. In colorectal-cancer patients who received adjuvant 5-FU chemotherapy, high MMP-7 tissue expression was found to be associated with shorter disease-free survival [25]. In BC, we previously found elevated MMP-7 mRNA, serum and urine levels to correlate with the presence of lymph node metastasis [26]. A further study by Bolenz et al. demonstrated that inhibition of MMP-7 resulted in decreased invasive capability of BC cells [27]. In the present study, we assessed the prognostic value of MMP-7 for prediction of OS and PFS in patients with MIBC who underwent platinum-based chemotherapy. Our data show that high MMP-7 tissue expression is significantly associated with shorter OS and tended to correlate with PFS. These correlations proved to be significant in the multivariate analysis, suggesting MMP-7 as an independent risk factor for PFS and OS in platinum-treated BC patients. Similar to the findings in tissue samples, we found that high MMP-7 concentrations in serum samples taken before platinum chemotherapy were independently associated with shorter OS. Accordingly, the median OS time in patients with high pretreatment serum MMP-7 serum level was 12.8 months, which was significantly shorter compared to 20.8 months in patients with low MMP-7 concentrations. This correlation remained independent of other clinicopathological parameters. 

Tissue IHC analysis allows for the detection of the cellular and subcellular distribution of MMP-7 staining, which may provide additional information. Therefore, it may be preferred if the time delay between surgery and planned chemotherapy is rather short. In such cases, when the planned chemotherapy follows previous surgery after more than three months, the use of serum samples may better reflect current status of MMP-7. 

Our tissue (IHC) and serum (ELISA) cohorts were not overlapping, and therefore a direct comparison between tissue and serum MMP-7 levels was not possible. However, in a previous study, we assessed corresponding serum and tissue samples from the same BC patients and found a strong correlation between their MMP-7 levels. This suggests that high MMP-7 serum levels are directly associated with enhanced MMP-7 tissue expressions [26]. A further aspect that we could not address in this study is the possible circadian change of MMP-7 serum levels. However, MMP-7 concentrations in most of our follow-up samples from the same patients in the present and also in previously published studies were relatively stable, showing only rather small variations. Based on this, we speculate, that that circadian changes have most probably only little or no significant impact on MMP-7 serum levels.

Several studies suggested that MMP-7 plays a causal role in the development of platinum resistance in different tumor types [12,13,14,15,16]. In the present study, we determined the gene expression of *MMP-7* in a series of BC and normal urothelial cell lines. Overall, *MMP-7* was variably expressed in the investigated BC cell lines. We then stably transfected two BC cell lines (RT-112 and T-24) with low MMP-7 expression, but we could not observe a significant change in their platinum sensitivity. This may be explained by the observation that cisplatin resistance may originate from a combination of various different factors [22]. Thus, in vitro manipulation of one factor alone may not be enough to reverse resistance, so that we cannot completely rule out from our data a functional impact of MMP-7. Further studies with stable conditions, e.g., CRISPR/Cas9 knockout, will be needed in the future, to study the selection for resistant subclones over months or the development of drug-persister cells. Another possible explanation for this may originate from the immunohistochemical observation that enhanced MMP-7 expression is frequently localized to the tumor–normal and tumor–stoma interface, suggesting that an interaction with non-malignant cells is necessary for the expression of *MMP-7*. This association was further confirmed by our in silico analysis, showing a significant direct significant correlation between stromal and immune signatures and *MMP-7* gene expression. The interaction between stromal cells from the tumor microenvironment and BC cells with regard to mediation of cisplatin resistance should be investigated in the future, in more complex co-culture models.

However, the in silico analysis revealed no association between *MMP-7* gene expression and OS in the TCGA dataset. Considering that only a small subset of the TCGA cohort was reported to receive chemotherapy, this may suggest that the prognostic value of MMP-7 is rather related to the use of chemotherapy and so is rather predictive than prognostic. However, we formerly found a prognostic value for *MMP-7* gene expression [26]. Therefore, further research needs to be performed to assess the predictive value of MMP-7. 

Platinum-based therapies in advanced MIBC provide a response rate of approximately 50% associated with relatively high toxicity. As immune checkpoint and FGFR-inhibitors are now available for platinum-resistant MIBC patients, a preselection of platinum-resistant patients may help to reduce unnecessary side-effects and the early administration of potentially effective immune checkpoint or FGFR inhibitors. Our present data suggest that MMP-7 is a promising marker to select patients who will benefit from platinum-based chemotherapy; however, its predictive value has to be confirmed in independent prospective cohorts. 

In the last years, several potential therapy-predictive markers, such as the excision repair gene *ERCC1*, *HMGA2*, nucleoside transporter protein hENT1, transcription factor TFAP, apoptosis inhibitor surviving, EMMPRIN and SDC1, have been identified [28,29,30,31,32]. In addition, gene-expression-based molecular subtypes of MIBC were suggested to show different sensitivities to platinum therapy [8,9]. However, currently, none of these markers has become established in routine clinical practice. Considering that there are various molecular ways how cancer cells may acquire chemoresistance, we speculate that a combination of markers involved in different molecular pathways might provide the highest predictive accuracy. Further research has to decide which marker constellation offers the most accurate prognosis for platinum-treated MIBC patients.

## 5. Conclusions

Our present data revealed that high MMP-7 tissue expression and high pretreatment serum concentrations are independently associated with shorter OS in patients with platinum-treated MIBC. Therefore, if these findings can be confirmed in independent studies, MMP-7 analysis might help to select patients who will benefit from a platinum-containing chemotherapy, while other patients might be saved from unnecessary toxicity and may benefit from an early administration of other available therapies. Our in vitro analyses did not confirm the functional involvement of MMP-7 in platinum resistance. As our IHC and in silico analyses uniformly suggested the importance of stromal cells in the modulation of MMP-7 expression, we speculate that in vitro analyses with cancer monoculture may not be fully representative regarding the functional spectrum of MMP-7. Therefore, further research is needed to reveal the involvement of MMP-7 in platinum resistance of MIBC.

## Figures and Tables

**Table 1:** Patients’ characteristics.

Cohort	Serum MMP-7	Tissue MMP-7	*p*
Variables	*n* (%)	*n* (%)	
Total number of patients	52 (100)	72 (100)	
Age at baseline median (range)	65 (41–81)	64 (37–90)	
≤65	27 (52)	37 (51)	0.953
>65	25 (48)	35 (49)	
Sex			
Male	38 (73)	50 (69)	0.660
Female	14 (27)	22 (31)	
ECOG PS at enrollment			
0	43 (83)	37 (51)	**<0.001**
≥1	9 (17)	35 (49)	
Stage			
pT1	1 (2)	1 (1)	
pT2	9 (17)	16 (22)	
pT3	21 (40)	24 (33)	
pT4	10 (20)	13 (18)	
pT1–pT2	10 (19)	17 (23)	0.448
pT3–pT4	31 (60)	37 (51)	
not available	11 (21)	18 (26)	
Metastases			
Lymph node metastasis (>2 cm)	34 (65)	41 (72)	0.343
Distant metastasis	12 (23)	44 (61)	**<0.001**
Soft tissue lesions (lung/liver)	9 (17)	36 (50)	**<0.001**
Bone metastasis	3 (6)	11 (15)	0.145
Chemotherapy regimen			
Gem/Cis	47 (90)	68 (94)	0.471
Gem/Carbo	5 (10)	2 (3)	
MVAC	0 (0)	2 (3)	
Curative (prior cystectomy)	44 (85)	57 (79)	0.289
Palliative (no prior cystectomy)	8 (15)	15 (21)	
Number of cycles median (range)	3 (1–9)	4 (1–8)	
Collection site			
SUSE	0 (0)	43 (60)	
Essen	16 (31)	29 (40)	
Budapest	36 (69)	0 (0)	
Number of patients died	31 (60)	53 (74)	
Follow-up time in months median (range)	17 (2–101)	9 (1–102)	

MMP-7—matrix metalloproteinase-7; ECOG—Eastern Co-Operative Oncology Group; PS—performance status. Gem/Cis—gemcitabine + cisplatin, Gem/Carbo—gemcitabine + carboplatin, MVAC—methotrexate + vinblastine + doxorubicin + cisplatin. SUSE—a phase II, prospective, multicenter, randomized, double-blinded trial. Bold printed *p*-values were significant (<0.05).

**Table 2:** MMP-7 serum and tissue levels and clinicopathological parameters. Low MMP-7 tissue expression (score ≤ 3) and high MMP-7 tissue expression (score > 3).

Cohort	Serum MMP-7		Tissue MMP-7			
Variables	ng/mL	*p*	All Patients	Low MMP-7	High MMP-7	*p*
	Median (Range)		*n* (%)	*n* (%)	*n* (%)	
Total number of patients	52		72 (100)	53 (74)	19 (26)	
Age (range)	65 (41–81)		64 (37–90)			
≤65	7.31 (3.33–18.16)	0.473	37 (51)	24 (33)	13 (18)	0.083
>65	10.08 (3.12–21.11)		35 (49)	29 (40)	6 (8)	
Sex						
Male	9.01 (3.33–19.85)	0.983	50 (69)	39 (51)	11 (15)	0.203
Female	8.97 (3.12–21.11)		22 (31)	14 (19)	8 (11)	
Stage						
pT1–pT2	10.51 (6.68–17.63)	0.083	17 (31)	12 (22)	5 (9)	0.692
pT3–pT4	7.07 (3.12–19.85)		37 (69)	28 (52)	9 (17)	
not available			18	13	5	
Performance status						
0	8.73 (2.12–21.11)	0.956	37 (51)	28 (39)	9 (12)	0.683
≥1	9.26 (3.33–18.16)		35 (49)	25 (35)	10 (14)	
Visceral metastasis						
absent	7.55 (3.12–19.85)	0.315	32 (44)	26 (36)	10 (14)	0.789
present	10.27 (3.51–21.11)		40 (56)	27 (38)	9 (12)	
Distant metastasis						
absent	9.01 (3.12–21.11)	0.912	35 (65)	22 (31)	6 (8)	0.446
present	9.24 (3.33–18.16)		19 (35)	31 (43)	13 (18)	
not available			18			
Collection site						
SUSE	-		43 (60)	34 (47)	9 (13)	0.201
Essen	10.98 (3.12–17.63)	0.934	29 (40)	19 (26)	10 (14)	
Budapest	8.00 (3.33–21.11)		-			

**Table 3:** Cox univariate overall survival (OS) and progression-free survival (PFS) analysis in the enzyme-linked immunosorbent assay (ELISA) and immunohistochemistry (IHC) cohort.

Univariate Analysis	Serum MMP-7						Tissue MMP-7					
Variables	Overall Survival			Progression-Free Survival			Overall Survival			Progression-Free Survival		
	HR	95% CI	*p*	HR	95% CI	*p*	HR	95% CI	*p*	HR	95% CI	*p*
Age												
≤65	ref.			ref.			ref.			ref.		
>65	1.119	0.553–2.266	0.755	0.403	0.142–1.145	0.088	1.315	0.760–2.275	0.328	1.242	0.746–2.065	0.405
Sex												
Male	ref.			ref.			ref.			ref.		
Female	0.648	0.266–1.581	0.340	0.934	0.303–2.877	0.906	1.414	0.771–2.595	0.263	1.116	0.628–1.984	0.708
Stage												
pT1–pT2	ref.			ref.			ref.			ref.		
pT3–pT4	0.659	0.279–1.558	0.342	0.760	0.234–2.474	0.649	1.109	0.563–2.187	0.764	1.406	0.719–2.751	0.320
Performance status												
0	ref.			ref.			ref.			ref.		
≥1	1.391	0.703–2.757	0.343	1.641	0.804–3.349	0.173	2.278	1.298–3.999	**0.004**	2.098	1.247–3.529	**0.005**
Lymph node status												
N0/Nx	ref.			ref.			ref.			ref.		
N+	0.632	0.305–1.307	0.216	1 469	0.475–4.542	0.504	0.61	0.349–1.066	0.083	0.672	0.399–1.131	0.134
Distant metastasis												
absent	ref.			ref.			ref.			ref.		
present	2.459	1.147–5.273	**0.021**	7.832	2.627–23.349	**<0.001**	1.648	0.911–1.648	0.099	1.276	0.737–2.162	0.364
Visceral metastasis												
absent (only N+)	ref.			ref.			ref.			ref.		
present	2.862	1.252–6.544	**0.013**	5.471	1.870–16.010	**0.002**	1.584	0.910–2.756	0.104	1.402	0.842–2.336	0.194
Serum MMP-7 (median)												
<10.0 ng/mL	ref.			ref.			-			-		
>10.0 ng/mL	2.176	1.045–4.530	**0.038**	1.704	0.652–4.451	0.277	-	-	-	-	-	-
Clinical response												
CR, PR, SD	-			-			ref.			ref.		
PD	-	-	-	-	-	-	5.295	2.726–10.285	**<0.001**	6.255	3.272–11.957	**<0.001**
Tissue MMP-7 expression												
low ≤3	-			-			ref.			ref.		
high >3	-	-	-	-	-	-	2.061	1.125–3.778	**0.019**	1.613	0.912–2.851	0.100

HR—hazard ratio, CI—confidence interval, ref.—referent. Bold-printed *p*-values were significant (<0.05).

**Table 4:** Cox multivariate OS and PFS analysis in the ELISA and IHC cohort.

Multivariate Analysis	ELISA Cohort (Serum)					
Variables	Overall Survival			Progression-Free Survival		
	HR	95% CI	*p*	HR	95% CI	*p*
Distant metastasis						
absent	ref.			ref.		
present	1.090	0.248–4.794	0.909	0.826	0.155–4.405	0.823
Visceral metastasis						
absent (only N+)	ref.			ref.		
present	3.483	0.669–18.119	0.138	10.006	1.768–57.629	**0.009**
Serum MMP-7 (median)						
<10.0 ng/mL	ref.			ref.		
>10.0 ng/mL	2.743	1.258–5.984	**0.011**	2.061	0.762–5.575	0.154
ECOG Performance status						
0	ref.			ref.		
≥1	2.455	1.386–4.348	**0.002**	2.228	1.314–3.777	**0.003**
MMP-7 expression						
low ≤3	ref.			ref.		
high >3	2.296	1.235–4.268	**0.009**	1.791	1.003–3.198	**0.049**

HR—hazard ratio, CI—confidence interval, ref.—referent. Bold-printed *p*-values were significant (<0.05).

## Data Availability

Publicly available datasets were analyzed in this study. This data can be found here: [(http://www.cbioportal.org/study?id=blca_tcga)]. Further own experimental data is available on request from the corresponding author.

## Supplementary Materials

The Supplementary Materials are available online at https://www.mdpi.com/2075-4418/11/1/48/s1. Materials and Methods to cell culture and cell experiments. Figure S1: Changes of MMP-7 levels during platinum therapy. Curves represent MMP-7 serum levels of patients without disease progression (A) and progression (B) or different survival times (C,D) at time points during chemotherapy cycles.

## Abbreviations

BC	bladder cancer
CI	confidence interval
ECM	extracellular matrix
ECOG PS	Eastern Co-Operative Oncology Group performance status
EMT	epithelial-to-mesenchymal transition
FFPE	formalin-fixed and paraffin-embedded
FGFR	fibroblast growth factor receptors
GC	gemcitabine + cisplatin
HNSCC	head and neck squamous cell carcinoma
HR	hazard ratio
IHC	immunohistochemistry
MIBC	muscle-invasive bladder cancer
MMP-7	matrix metalloproteinase-7
MVAC	methotrexate + vinblastine plus + doxorubicin + cisplatin
OS	overall survival
PFS	progression-free survival
UCCs	urothelial carcinoma cells
