# Supplementary material for: MMP-7 Serum and Tissue Levels Are Associated with Poor Survival in Platinum-Treated Bladder Cancer Patients
Source: Diagnostics (Basel). 2020 Dec 31;11(1):48. doi: 10.3390/diagnostics11010048 (PMC7824149; doi:10.3390/diagnostics11010048)
Supplement: Supplementary file 1 [file diagnostics-11-00048-s001.pdf]

Supplementary Materials and Methods

Cell Culture and cell experiments

Urothelial carcinoma cells (UCC) were cultured at 37 °C and 5% CO_2 in DMEM GlutaMAX-I (Gibco, Darmstadt, Germany) supplemented with 10% fetal calf serum (FCS, Biochrom, Berlin, Germany), except for KU-19-19 which was cultured in RPMI-1640 (Gibco). As normal controls, we used spontaneously immortalized uroepithelial HBLAK cells [18], kindly provided by the CELLnTEC company and TERT-NHUC, a culture of primary urothelial cells (NHUC), which was immortalized by stable overexpression of human telomerase reverse transcriptase (hTERT), kindly provided by Dr. Knowles (Leeds, UK) [19]. TERT-NHUC cells were cultured in keratinocyte serum-free medium (Gibco) supplemented with 0.35 µg/ml N-epinephrine and 0.33 mg/mL hydrocortisone. HBLAK cells were cultured according to the manufacturer's recommendation in serum-free CnT-Prime Epithelial Culture Medium (CELLnTEC, Bern, Switzerland).

Gene expression analysis

For gene expression analysis RNA extraction was performed using the RNeasy Mini Kit according to the manufacturer (Qiagen, Hilden, Germany). One µg RNA was reverse transcribed into cDNA using the QuantiTect Reverse Transcription Kit (Qiagen), with an extended incubation time of 30 min at 42◦C. Quantitative reverse transcription polymerase chain reaction (qRT-PCR) was performed with Luna® Universal qPCR Master Mix (New England Biolabs, Frankfurt, Germany) according to the manufacturer's instructions on the LighCycler 96® platform (Roche, Grenzach-Wyhlen, Germany). MMP-7 expression was measured using primers MMP-7_fwd 5′-GAACGCTGGACGGATGGTAG- 3′ and MMP-7_rev 5′- TTTGGGGATCTCCATTTCCA- 3′. The housekeeping gene TATA-box binding protein (TBP) was used as a reference gene with the following primers:
TBP_fwd 5′- GAGCCAAGAGTGAAGAACAGTC- 3′and
TBP_rev 5`- GCTCCCCACCATATTCTGAATCT- 3′.
qRT-PCR was performed using initial denaturation at 95 ◦C for 2 min and 45 cycles of amplification including denaturation at 95◦C for 10s, annealing and elongation for 30s at 60°C and a melting curve analysis.

Stable overexpression of MMP-7 and analysis of cisplatin sensitivity

For the process of stable overexpression of MMP-7 empty vector was generated by excision of the MMP-7 cDNA sequence using restriction enzymes EcoRI and XbaI (New England Biolabs, Frankfurt, Germany). RT-112 and T-24 cells were transfected using X-tremeGENE™ 9 according to the manufacturer (Merck, Darmstadt, Germany) and selected with G418 (Sigma-Aldrich, Taufkirchen, Germany). Stable overexpression was confirmed by qRT-PCR. Stably overexpressing MMP-7 UCC and vector control cells were treated with a single dose of indicated final concentrations of cisplatin for 72h. Cell viability was then measured in quadruplicates by means of CellTiter-Glo assay (Promega, Fitchburg, WI, USA).

Supplementary figure

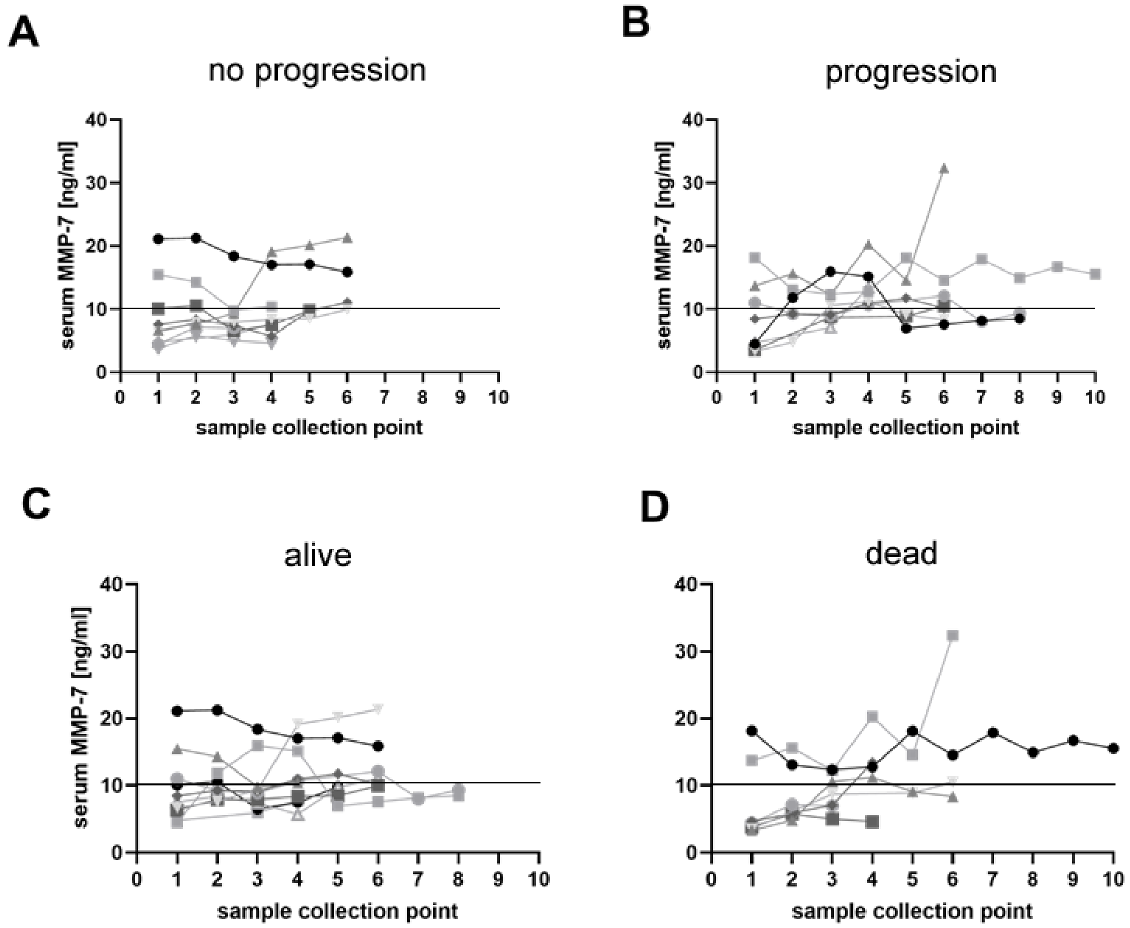

Figure S1. Changes of MMP-7 levels during platinum therapy. Curves represent MMP-7 serum levels of patients without disease progression (A) and progression (B) or different survival times (C, D) at time points during chemotherapy cycles. Horizontal lines represent the 10 ng/mL MMP-7 concentration, the cut-off value of high MMP-7 serum level before chemotherapy. Serum samples were collected on every 3rd and 8th days of every chemotherapy cycles.
